# Epigenetics in the Diagnosis and Therapy of Malignant Melanoma

**DOI:** 10.3390/ijms23031531

**Published:** 2022-01-28

**Authors:** Simeon Santourlidis, Wolfgang A. Schulz, Marcos J. Araúzo-Bravo, Daniela Gerovska, Pauline Ott, Marcelo L. Bendhack, Mohamed Hassan, Lars Erichsen

**Affiliations:** 1 Epigenetics Core Laboratory, Institute of Transplantation Diagnostics and Cell Therapeutics, Medical Faculty, Heinrich-Heine University Duesseldorf, 40225 Duesseldorf, Germany; Simeon.Santourlidis@med.uni-duesseldorf.de (S.S.); pauline.ott@uni-duesseldorf.de (P.O.); 2 Department of Urology, Medical Faculty, Heinrich-Heine University Duesseldorf, 40225 Duesseldorf, Germany; Wolfgang.Schulz@uni-duesseldorf.de; 3 Group of Computational Biology and Systems Biomedicine, Biodonostia Health Research Institute, 20014 San Sebastián, Spain; mararabra@yahoo.co.uk (M.J.A.-B.); daniela.gerovska@biodonostia.org (D.G.); 4 IKERBASQUE, Basque Foundation for Science, 48009 Bilbao, Spain; 5 Department of Urology, University Hospital, Positivo University, Curitiba 80030-200, Brazil; marcelo@uro-onco.net; 6 Department of Surgery, Tulane University School of Medicine, New Orleans, LA 70112, USA; dr.hassan@gmx.de; 7 Institut National de la Santé et de la Recherché Médicale, University of Strasbourg, 67000 Strasbourg, France; 8 Institute for Stem Cell Research and Regenerative Medicine, Medical Faculty, Heinrich-Heine University Düsseldorf, 40225 Duesseldorf, Germany

**Keywords:** melanoma, DNA methylation, epigenetics

## Abstract

Epigenetic mechanisms are fundamentally important for cancer initiation and development. However, a survey of the literature reveals that, to date, they appear less comprehensively investigated in melanoma than in many other cancers, e.g., prostate, breast, and colon carcinoma. The aim of this review is to provide a short summary of epigenetic aspects of functional relevance for melanoma pathogenesis. In addition, some new perspectives from epigenetic research in other cancers with potential for melanoma diagnosis and therapy are introduced. For example, the PrimeEpiHit hypothesis in urothelial carcinoma, which, similarly to malignant melanoma, can also be triggered by a single exogenous noxa, states that one of the first steps for cancer initiation could be epigenetic changes in key genes of one-carbon metabolism. The application of such insights may contribute to further progress in the diagnosis and therapy of melanoma, a deadly type of cancer.

## 1. Introduction

One early starting point of an organism’s life is the pluripotent stem cell of the inner cell mass, which already harbors all needed inherited, evolutionary-shaped genetic, and epigenetic information. During later development, depending on which differentiation path is taken, only an adequate part of this information will come into practice, to fulfill the specialized demands on cellular function requested by the environment of each specifically differentiated cell. Thus, firstly, it is fascinating that all environmentally imposed information is burned within one undifferentiated cell, and secondly, that later it is selectively unleashed in a programmed and preparatory manner to determine differentiation and function of a cell in full compliance with the upcoming, multifaceted environmental needs. The precision of differentiation is the key to life.

During ontogeny, owing to the efforts of C. H. Waddington, we started to understand that this occurs through the gradual establishment of various epigenetic mechanisms, which build up a mediating bond between the genome and the environment and are heritable and plastic. This ensures the propagation of the unaltered cell functionality after division and the ability to adequately adapt to fluctuant environmental pressures, in an evolved space of tolerance, without losing the functional assignment.

In aging, we observe the opposite process, i.e., the gradual loss of the integrity of these epigenetic mechanisms accompanied by loss of cell function and cellular deterioration, followed by a substantial increase in the risk for cancer development. Obviously, a cancer cell has been forced to give up the original differentiation status and functional specificity and has reduced its existence to simple survival, often linked to an aberrant, out-of-joint elevated, unlimited proliferation rate and dedifferentiation that is reminiscent of stem cells. We see that this occurs because of pressuring harmful exogenous noxae. Since we can trigger them, obviously we are able to willingly impact our own epigenetics, and thus, we may, to some extent, master our fate.

The first epigenetic hallmark of cancer recognized since 1914, originally described by Theodor Boveri [1], is the reconfiguration of chromatin providing dense “hyperchromatic” sites, widely exploited diagnostically later, e.g., by George Papanicolaou in the 1930s [2], to save millions of lives [3]. Meanwhile, the main mechanisms involved in this are distinct combinations of histone modifications associated with active and non-active chromatin compartments, restructuring the genome-wide chromatin compaction and DNA methylation [4].

Firstly, described in 1950 [5], DNA methylation has been intensively investigated, especially in the last 35 years, and newly developed and adjusted methods have meanwhile reached a high standard of improvement; once concerning screening, their widespread applications now include histone modification-/DNA methylation array technology, bisulfite sequencing, and especially DNA methylation-specific PCR (MSPCR) for fast and cost-effective diagnosis, as well as the first FDA-approved epigenetic method for early detection of colon cancer.

## 2. Incidence and Etiology of Cutaneous Melanoma

Cutaneous Melanoma (CM) is an aggressive and invasive cancer of the skin, with 324,635 new cases and 57,043 new deaths in 2020 worldwide, contributing 1.7% of all cancers in the world population [6], with rising incidence [7]. Its incidence also increased with age, with new cases most common in the 65–74 age group [8]. The median age of onset is 64 years [8]. Melanoma causes severe morbidity since it often metastasizes at very early stages and often defies therapy by pronounced chemoresistance. The median survival of metastatic melanoma patients is 5–8 months [7]. The prognosis is even worse if melanoma affects mucosal areas [9].

Of all cancers, melanoma has the highest mutation frequency, with up to 100 mutations per Mb [10]. Its broad mutation landscape includes prominently genes involved in MAPK signaling. In 40–60% of all cutaneous melanomas, the *BRAF* proto-oncogene, encoding a serine–threonine kinase, is affected by activating point mutations, most commonly V600E [10]. In 28–30% of all melanomas, somatic mutations activate members of the proto-oncogene RAS GTPase family, primarily *NRAS*, followed by *KRAS* and *HRAS* [11]. Moreover, 13–17% of all melanomas, with loss-of-function mutations, inactivate the tumor suppressor Neurofibromin 1 (*NF1*) [11], a negative regulator of RAS signal transduction. Further important mutations in melanomas inactivate *CDKN2A* (13.3%) and *TP53* (15.1%), which are believed to allow oncogene-driven melanocytes to overcome senescence and evade apoptosis [12]. Interestingly, *CDKN2A* gene mutations inactivating the cell cycle inhibitor *p16* have been reported to be more frequent in metastases (14%) than in primary tumors (7%) [13]. It is assumed that the high variability in the mutation frequency in melanoma is attributed to whether the external inducer ultraviolet radiation (UV) was involved or not. More than 70% of cutaneous melanomas are believed to be caused by UV radiation exposure [11].

The differentiated, functionally highly specialized, pigment-producing melanocyte in the basal layer of the skin epidermis is persistently exposed to an exogenous noxa—namely, UV radiation. UVB, with wavelengths between 290 nm and 320 nm, is most harmful to the genome, causing prominently the formation of dimeric photoproducts between adjacent pyrimidine bases on the same strand, but also inducing a much wider range of DNA damage, such as protein–DNA crosslinks, single-strand breaks, and thymine glycol formation [14]. As a result, ultimately, irreparable damage of the DNA may force a choice between programmed cell death, apoptosis, or the transformation to a new, more dedifferentiated cell status that remains viable but may lose some or all of its specialized functions. This transformation is accompanied by extensive genetic mutations and chromatin restructuring processes.

## 3. Histone Modifications and Significance for Melanoma Progression

Multiple histone modification changes have been reported in CM, and some of them have been linked to tumor behavior. First, an increase in the global levels of demethylated histone H3 at lysine 9 (H3K9me2) has been found in melanoma samples, compared with the normal peritumoral skin tissue [15]. H3K9me2 recruits the heterochromatin protein 1 (HP1) which directs DNA methyltransferase 1 (DNMT1)-dependent DNA methylation in vivo [16] and plays a key role in the formation of transcriptionally inactive heterochromatin [17]. This histone modification is established by the histone methyltransferase G9a [16], which has been found significantly upregulated in melanoma patients [18] who have a poorer outcome [19]. Conversely, G9a silencing elevates the self-renewal capability of differentiated melanoma cells in a Sox2-dependent manner [20]. Sox2 is a master regulator of pluripotency in embryonic stem cells. In addition, inhibition of G9a induced cell death in diverse melanoma cell types and diminished tumor growth in vivo [21].

The importance of chromatin-modifying enzymes in regulating tumorigenesis was underscored in a zebrafish melanoma model. Overexpression of either SET domain bifurcated histone lysine methyltransferase 1 (SETDB1) or suppressor of variegation 3–9 homolog 1 (SUV39H1), both enzymes methylating histone H3 on lysine 9 (H3K9), significantly accelerates melanoma formation [22]. Independently, it was shown that the H3K9-specific methyltransferase SUV39H1 establishes trimethylation at H3K9 at the promoter of the tumor suppressor gene retinoblastoma 1 (*RB1*), and this recruits DNA methyltransferase 3A, which mediates DNA methylation of the promoter. Thus, RB1 expression becomes epigenetically repressed, and, in turn, E2F1, which is inhibited by RB1, becomes transcriptionally activated. The authors show that this promotes UV-induced skin tumorigenesis in vivo. Conversely, depletion of SUV39H1 in melanoma cells leads to RB1 activation and reduced E2F1 transcriptional activity, inhibiting melanoma development [23].

The RB protein exerts tumor suppressor function by negative control of the cell cycle and by binding to E2F family members and repressing their functions at the promoters of genes, which are important for S-phase progression and cell proliferation [24]. RB interacts with histone acetyltransferases (HATs) and -deacetylases (HDACs), supports repair of DNA double-strand breaks (DSB), chromosome condensation, and silencing of repetitive sequences. Through its chromatin regulatory functions, it affects genomic stability [24]. E2F1 promotes cell proliferation but only develops its oncogenic properties when pathways that mediate E2F1-induced apoptosis are disabled [25]. A variety of functional studies show that melanoma cells reprogram their survival pathways and expand their intrinsic resistance to apoptosis during melanoma progression [26]. Interestingly, the p16INK4A–Rb–E2F pathway, which is an important regulator of cell cycle and differentiation, and its dysfunction can lead to oncogenesis, is altered in more than 80% of human neoplasias [25]. p16INK4A arrests the cell cycle in G1 by inhibiting CDK4 and CKD6, thereby preventing the inactivation of pRB [27]. In 72 melanomas greater than 1.0 mm and 29 metastases, its expression has been found lost in 100% of the cases [28]. Straume et al. reported the loss of p16 protein expression by promoter hypermethylation in 19% of primary cutaneous melanomas and in 33% of metastases [29].

The RB1-target E2F1 positively regulates the enhancer of zeste homolog 2 (EZH2), which is a histone–lysine N-methyltransferase enzyme and a core subunit of the polycomb repressive complex PRC2, which is responsible for global changes of chromatin architecture and essential in early development but is downregulated in normal adult tissues. EZH2 is an important driver of melanoma progression [30], and its increased activity leads to increased global H3K27me3. Increased H3K27me3 is an indicator of poor prognosis and is associated with aggressive and metastatic forms of melanoma [31]. The 5-year survival rate of EZH2-high patients was 48%, compared with 71% in the EZH2-low group [32]. The EZH2-mediated elevation of H3K27me3 has been described to be involved in epigenetic silencing of the tumor suppressor genes RUNX3 and CDH1 in advanced-stage human melanoma tissues [33].

Notably, it has been proposed that abnormally high levels of EZH2 found in cancer cells may shift expression profiles toward a stem-cell-like state [30]. In melanoma, elevated EZH2 contributes to a shift to a more invasive and metastasizing phenotype. EZH2 inhibition was suggested as a promising approach for preventing the transition to advanced cancer stages [34]. Accordingly, pharmacological inhibition of EZH2 by GSK503 decreased melanoma progression in melanoma-bearing mice in vivo and doubled the survival time [35].

## 4. DNA Methylation Alterations in Melanoma

A further epigenetic hallmark of melanoma—namely, DNA methylation alterations, has been recently comprehensively reviewed [36]. In addition to focal DNA hypermethylation of the well-investigated tumor suppressor genes *PTEN*, *CDKN2A (p16/p14)*, and *RASSF1A*, with promoter hypermethylation prevalence of 6–62% (*PTEN*), 5–27% (*p16*), 41–57% (*p14*), and 15–57% (*RASSF1A*), respectively [36], focal hypermethylation of many more genes has been reported in melanomas. For instance, *COL1A2*, *NPM2*, *HSPB6*, *DDIT4L*, and *MT1G* promoter methylation was increased in eight early passage human melanoma cell lines/tissues, compared with newborn and adult melanocytes [37]. The validated markers were with a significant increase in methylation in advanced-stage melanomas. In another study of 16 melanoma cell lines, an elevated methylation status was reported for the following gene promoters: *ESR1* (50%), *MGMT* (50%), *RARB2* (44%), *RIL* (88%), *RASSF1A* (69%), *PAX7* (31%), *PGRB* (56%), *PAX2* (38%), *NKX2-3* (63%), *OLIG2* (63%), *HAND1*(63%), *ECAD* (88%), *CDH13* (44%), and *CDKN2A/p16* (6%) [38]. Conversely, the genes *CD2*, *EMR3*, *CARD15*, *EV12A*, *HLA-DP1*, *IFNG*, *IL2*, *ITK*, *KLK10*, *LAT*, *MPO*, *PSCA*, *PTHLH*, *PTHR1*, *RUNX3*, and *TNFSF8* have been found hypomethylated in 25 primary melanomas, compared with 29 benign nevi [39]. Interestingly, distinct hypermethylated genes have been found associated with genetic mutation subgroups, e.g., *NF1* hypermethylation with *NF1*- and *RAS*-mutated melanomas, *PTEN* hypermethylation with *BRAF*-mutated, and *CDK2A/B* hypermethylation with *BRAF*-, *RAS*-, *NF1*-mutated and triple-WT melanomas [40].

Repetitive LINE-1 retrotransposon hypomethylation may result in their reactivation, LINE-1 RNA, and protein expression, and has been linked to apoptosis, DNA damage and repair, tumor progression, cellular plasticity, and stress response [41]. In 75% of 16 melanoma cell lines, significant hypomethylation of LINE-1 sequences was found [42]. In a study comprising 46 primary melanomas and a 5-year follow-up period, LINE1 hypomethylation was reported to be accompanied by a shortened relapse-free survival of the patients and to be significantly associated with metastasis [42]. In a further study, LINE-1 retroelements were assessed in resected melanoma tissues from 82 patients ranging in age from 14 to 88 years. LINE-1 methylation was found decreased in melanoma patients with clinical parameters associated with an adverse prognosis [43].

## 5. One-Carbon Metabolism in the Etiology of Epigenomic Aberrations in Melanoma

While a plethora of DNA methylation alterations occur in melanoma, it remains elusive how they are caused. It has been proposed that either active processes, e.g., an aberrant activity or function of DNMT enzymes, or passive ones, for instance, changes in epigenetic modifications that regulate targeting of DNA methylation, may be involved [36].

Based on our recent observation in urothelial carcinoma (UC) [44,45], we would like to point out a further passive mechanism that may also apply to melanoma. A major risk factor for bladder cancer is persistent exposure to the harmful carcinogens of tobacco smoking, which is estimated to account for 50% of tumors [44] and contributes to the high mutational rate in that cancer. There is thus a parallel to the etiology of melanoma, where exposure to another exogenous carcinogen—harmful UVB radiation—is the major cause. The previously proposed PrimeEpiHit hypothesis for UC [44,45] may, therefore, explain methylation alterations in CM carcinogenesis. According to this modified hypothesis, chronic UVB radiation exposure may occasionally also hit genes with key functions in one-carbon-group metabolism. As a result, their transcription may become impaired, and subsequently, their epigenetic status may be altered. Epigenetic silencing because of gene disruption has been experimentally demonstrated [46]. Interestingly, analyzing a comprehensive mortality rate dataset for 30 types of cancer for 52 provinces in Spain, spanning 1978–1992, it has been found that melanoma correlated with bladder and lung cancer, suggesting common risk factors and mechanisms [47]. Key genes involved in one-carbon-group metabolism of course comprise a very small percentage of the whole genome, but due to the chronic carcinogen exposure, nonetheless, this may occur at some minor frequency, which appears in accordance with the low incidence.

**Figure 1:**
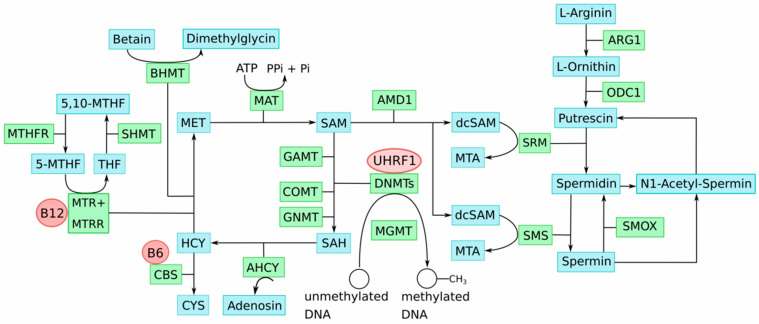
“One-carbon metabolism” [49,50] including the polyamine metabolic pathway [51]. Metabolites: 5-MTHF = 5-methyl tetrahydrofolate, 5,10-MTHF = 5,10-methylene tetrahydrofolate, CYS = cystathionine, dcSAM = decarboxylated S-adenosyl-L-methionine, HCY = homocysteine, MET = methionine, MTA = methylthioadenosine, SAM = S-adenosyl methionine, SAH = S-adenosyl homocysteine, THF = tetrahydrofolate. Enzymes: AHCY = adenosyl homocysteinase, AMD1 = adenosyl methionine decarboxylase, ARG1 = arginase 1, BHMT = betaine homocysteine S-methyltransferase, CBS = cystathionine ß-synthase, COMT = catechol-O methyltransferase, DNMT = DNA methyltransferase, GAMT = guanidinoacetate N-methyltransferase, GNMT = glycine methyltransferase, MAT = methionine adenosyl transferase, MTR = methionine synthase, MTHFR = methylene tetrahydrofolate reductase, MTRR = methionine synthase reductase, ODC1 = ornithine decarboxylase 1, SHMT = serine hydroxymethyl transferase, SMOX = spermine oxidase, SMS = spermine synthase, SRM = spermidine synthase Co-factors: B6 = vitamin B6, B12 = vitamin B12, UHRF1 = ubiquitin-like with PHD and ring-finger domains 1.

Impairment of key genes of one-carbon-group metabolism causes imbalances in the involved methyl group metabolic pathways. This disturbs the delicate SAM:SAH ratio and, consequently, genome-wide DNA methylation alterations, including LINE-1 hypomethylation that contributes to genetic instabilities; thus, cellular transformation occurs. Notably, this process could be enhanced by the well-described deficiencies of one-carbon-group metabolism associated with aging, which are likewise characterized by accumulation of SAH and DNA hypomethylation [48]. Age is an important risk factor for UC, as well as CM. Figure 1 provides an overview illustration of the key metabolites and enzymes involved in methyl group and polyamine metabolism and their interactions.

The PrimeEpiHit hypothesis assumes that, e.g., imbalances in polyamine biosynthesis cause deficiencies in methyl group levels in proliferating cancer cells. Specifically, aberrantly increased methylation of the ODC1 gene in proliferating cells leads to trapping of SAM as decarboxylated S-adenosylmethionine (dcSAM), which inhibits methylation reactions and impedes the supply of methyl groups for DNA methylation [52]. In support of this hypothesis, we demonstrated that the 5′-regulatory region of *ODC1* is hypermethylated in most analyzed early UC (pTa/pT1) specimens and that its promoter activity can be efficiently repressed by DNA methylation [45]. Moreover, *ODC1* gene repression in uroepithelial cells by RNA interference in vitro induced LINE-1 demethylation, LINE-1 transcriptional activation, and DNA double-strand breaks [45].

Table 1 presents an overview of expression data for key genes in one-carbon-group metabolism and DNA methyltransferases in melanoma based on the results of the comprehensive TCGA study [53]. While these results require confirmation in independent studies, ODC1 is downregulated in melanoma, compared with controls as well. In contrast, ODC1 expression is upregulated in many other cancer types, e.g., prostate, colon, and esophageal carcinoma show an elevated expression [53].

Thus, *ODC1* DNA methylation status, the potential impairment of its activity, and its potential role for cancer initiation and propagation should be investigated in melanoma.

In addition, an extended examination of further epigenetic disturbances of key factors of the one-carbon-group metabolism, e.g., *FOLRs*, *GNMT*, *MGMT*, etc., influencing the epigenome may reveal new potential targets for diagnosis and prognosis of CM.

## 6. Epigenetic Diagnosis Based on Differential Chromatin Organization and DNA Methylation in Melanoma

Sensitive and specific biomarkers are urgently needed for early diagnosis and classification, prognosis, and risk assessment to optimize individualized therapeutic choices in melanoma. If apparently complete remission is achieved by therapy, modern and meaningful biomarkers are needed to support the decision to stop treatment at the earliest and the right point, to concomitantly minimize the risk of recurrence and avoid the adverse events associated with unnecessary prolonged therapeutic treatment. As partly summarized above, distinct melanoma phenotypes could be driven by distinct aberrations of their epigenome. It is believed that epigenetic alterations exist in cancer, within the chromatin organization, and within the methylome, which comprises all methylated CpG dinucleotides, consistent within a distinct cancer cell population or even few that may be largely consistent between various cancer entities, opening new perspectives for pan-cancer diagnosis.

Focusing first on cancer-specific differential chromatin organization, for taking advantage of it in cancer diagnosis and therapy, we suggest screening after sites of chromatin with distinct differential organized structures for various melanoma stages, e.g., the first mesenchymal stage, which arises after the epithelial–mesenchymal transition. Once we detect such promising chromatin areas with, e.g., a more relaxed chromatin organization in a pathogenic melanoma cell population, we would be able to target it either for diagnosis or even for therapy. For diagnosis, e.g., taking advantage of the limited effectiveness of micrococcus nuclease (MN) on closed chromatin, it would be possible to pretreat melanoma and reference DNA and provide cancer-cell-originated DNA templates of MN-affected integrity for assaying them by sensitive PCR assays, as previously demonstrated [55]. For therapy, those cancer-cell-specific, differential organized chromatin areas could be targeted by CRISPR/Cas in vivo, taking advantage of its selective preference feature. Evolved as an adaptive immunity system that protects bacteria and archaea against phages and plasmids [56] by directing sequence-specific Cas9 endonuclease-mediated double-strand DNA cleavage (DSB) to the intruder’s DNA, and hence destroying its genetic information [57], this system has evolutionarily never been encountered as a substrate DNA, which is organized in complex, high-order, structured chromatin and, hence, has a high preference for binding to more easily accessible chromatin regions [58,59,60]. Among others, interesting concrete targets suggested here would be the genome-wide-distributed LINE-1 retrotransposons, which are densely methylated at their CpG dense promoter regions, tightly packaged into inactive chromatin, and poorly accessible for transcription in healthy differentiated somatic cells [61]. On the other hand, LINE-1 hypomethylation and activation is a common phenomenon in many different cancers [62,63], interestingly associated with cancer progression, becoming more pronounced in high-stage and high-grade cancer [64,65], and it is established that activation prevents chromatin compaction [66]. These new perspectives concerning the mentioned epigenetic alterations and LINE-1 should be considered and studied for diagnosis and therapy in melanoma.

**Figure 2:**
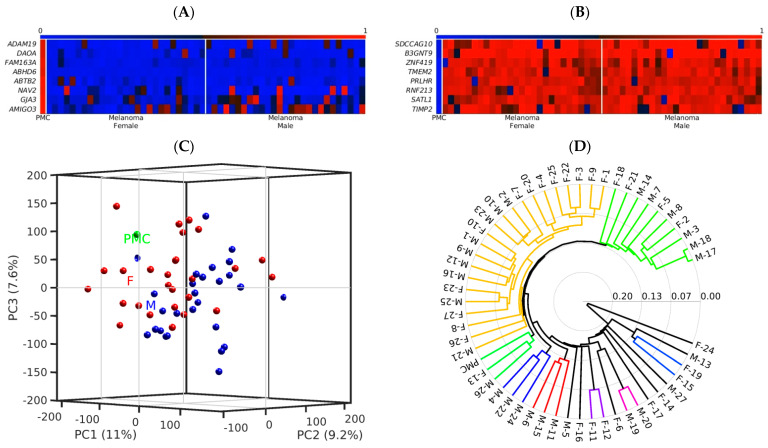
Bioinformatics analysis of DNA methylation microarray data from female and male melanoma samples and primary human melanocytes (PMC) [68]. We identified many short CpG-rich DNA fragments (60nt), which are consistently differentially methylated in almost all cancer samples (27 female and 27 male) by direct comparison with the control, in this case, PMC, both unmethylated (**A**) and methylated (**B**). This approach reveals differential DNA methylation based on every single array-probe (blue squares, hypomethylated; red squares, hypermethylated) consisting of 2–3 differentially methylated CpG dinucleotides and provides a new valuable level of resolution of DNA methylation analyses in addition to the classical CpG island analysis. Based on all 400.000 CpG probes analyzed for every single sample, we were able to perform Principal component analysis (PCA) (**C**) and subgroup classification (**D**) and name the relevant genes. At global DNA methylomics level, the female (F) and male (M) melanoma samples mix with each other (Figure 2C).

A further basic approach we pursued is to find within a certain distinct cancer cell population of interest, differentially methylated CpG dinucleotides forming a distinct, consistent, and characteristic methylation signature for this cell population. In our former investigations on prostate cancer and urothelial cancer specimens from patients, we were able to discover such unique DNA methylation signatures. The proposed strategy [45] is to screen using DNA methylation array technology after such differentially methylated CpG regions, and once detected, to validate and precisely dissect the cancer cell-specific DNA methylation pattern by bisulfite genomic sequencing [67]. Afterward, based on this information, we can define the outmost most suitable and robust MSPCR primers to sensitively detect this methylation signature. In Figure 2A,B, we show an example of how clear this differential methylation occurs between cancer and reference samples when the analysis is based on only a few CpG dinucleotides covered by the array probes, instead of surveying whole CpG islands, due to their implied functional relevance. In all cancer samples, we uncovered many consistently hypomethylated (blue) and hypermethylated (red) CpG-rich probes associated with the named genes. To illustrate this, we decided to choose independent data, recently published by others [68]. With respect to the role of epigenetic aberrations in melanoma, the authors reported in their study that a hierarchical clustering analysis provides stratification into two DNA methylation epigenotypes with high- and low-methylation subgroups. Interestingly, the prognosis was significantly worse in high-methylation cases [68]. Our own bioinformatic analysis of these publicly available data (GEO database under the accession number GSE140171 (GSM4155680-GSM4155734)) independently confirmed and extended this methylome-based stratification to more subgroups, as shown in Figure 2D. This subgrouping remains to be validated and correlated with the clinical outcome, to estimate its potential for prognosis.

Furthermore, we recently presented a new methodological approach to separate cell-free DNA from cellular DNA and unreservedly apply bisulfite genomic sequencing and MSPCR on this pure cell-free DNA. It is established that all tumors shed their cell-free DNAs that bear their unique DNA methylation patterns into the bloodstream [67], hence providing an easily accessible and exciting Achilles’ heel of cancer for effective diagnosis. For instance, PTEN promoter methylation was reported in cell-free DNA from 62% of the melanoma serum samples examined by pyrosequencing, indicating a good correlation with the same epigenetic alteration found in paired melanoma tissues [69]. Furthermore, LINE-1 hypomethylation, detected in both tissues and plasma-circulating DNA of melanoma patients, seems to be a hallmark of the metastatic capacity of primary melanomas, and LINE-1 hypomethylation may predict the overall survival in stage III CM patients [70].

MSPCR has been improved by us and enables, for the first time, relative quantification of DNA methylation in samples with identical and unequal genetic settings [71]. This is of importance for cancer samples, which often yield genetic aberrations, including melanoma, characterized by a higher number of chromosomal structural aberrations [72], and bladder carcinoma, characterized by early (pTa, pT1) chromosomal changes and imbalances [73]. A conventional normalization of MSPCR by using a housekeeping gene such as GAPDH or the ACTB gene may result in false results, especially if tumor samples from different patients must be compared, because, e.g., the copy number of the DNA segment containing the housekeeping gene chosen for normalization may vary in cancer DNA. The reliable measurement of DNA methylation by using MSPCR is based on DNA amplification, and it is extremely hindered by a variable number of template segments. In contrast, by using the idiolocal normalization of real-time, methylation-specific PCR (IDLN–MSP) [71], the normalization loci are chosen adjacent to the targeted loci. With this approach, it is guaranteed that in real-time MSPCR, the number of normalization loci is always as high as the number of targeted loci, reducing false-negative results in a significant manner, and this contributes to a significant improvement of DNA methylation measurements by MSPCR in tumor samples of different patient origin [71].

## 7. Conclusions

Surveying the known literature on melanoma genetics and epigenetics, one may easily infer that epigenetic restructuring is a major driver of melanoma development. Epigenetic changes are the only known alterations in cancer that hold the potential to be largely consistent in all relevant cells of a certain tumor stage. This is of high relevance for therapy and diagnosis. Additionally, we believe that, on that basis, even pan-cancer biomarkers will be defined in near future. For sure, it is yet not clear to which extent melanoma cell dedifferentiation resort to epigenetic programs of stem cells. Defining this fact may help us to better understand the nature of this deadly cancer and more successfully infer from our knowledge in stem cell research to target its vulnerability. Certainly, global, comprehensive epigenetic reorganization occurs. On the other hand, specific functionally relevant epigenetic reconfiguration of tumor suppressor genes and oncogenes also occurs. Focusing on the invasive and metastasizing melanoma cell population, it appears likely that one will be able to uncover specific chromatin and DNA methylation changes that exhibit consistent occurrence in all relevant cancer cells of the same stage. Thus, these may be preferred targets for therapy and diagnosis. Tumor-specific, epigenetically relaxed repetitive elements and other euchromatic regions may present new targets for CRISPR/Cas targeting to induce DSBs, taking advantage of the already disturbed DSB repair mechanisms. On the other hand, to widen the resolution of CpG differential methylation analyses on single, differentially methylated CpG sites holds the potential to reveal new consistently occurring differentially methylated sites for exploration in diagnosis. Here, one focus may be on cfDNA exhibiting melanoma cell-specific differentially methylated signatures relying on single, meaningful CpG-rich gene promoter regions, e.g., *RASSF1A*, LINE-1, or *ODC1.*

## 8. Material and Methods

The aim of this research was to provide a concise overview of epigenetic alterations in melanoma and provide evidence for their functional relevance in pathogenicity and/or possible suitability for their exploitation in diagnosis. We selected relevant publications on incidence and etiology of melanoma, epigenomics alterations of melanoma progression, including histone modifications and one-carbon metabolism, and epigenetic diagnosis based on differential analysis of epigenomics data. To perform our computational analysis, we downloaded publicly available DNA methylation data from female and male melanoma samples and primary human melanocytes (PMCs) from the Gene Expression Omnibus (GEO) database, under the accession number GSE140171, deposited by Yamamoto et al., as described in [68]. We analyzed the data by in-house-developed bioinformatics methods as described in [74,75].

## Figures and Tables

**Table 1:** **TCGA data for skin cutaneous melanoma (SKCM)**. Fold change in comparison with healthy controls [53]. The samples consisted of 67 (20%) primary cutaneous melanomas (all originating from non-glabrous skin) and 266 (80%) metastases [54].

Gene	Fold Change	Gene	Fold Change
*FOLR2*	0.38	*DNMT3a*	0.919
*GNMT*	0.471	*AHCYL1*	0.988
*BHMT2*	0.48	*MTHFR*	0.99
*MAT2B*	0.489	*DNMT1*	1.01
*ODC1*	0.533	*BHMT*	1.08
*SHMT1*	0.559	*MAT2A*	1.11
*MGMT*	0.612	*AHCYL2*	1.13
*TCN2*	0.654	*SMS*	1.34
*AMD1*	0.706	*AHCY*	1.52
*UHRF1*	0.877	*SMOX*	1.72
*DNMT3b*	0.893	*ARG1*	2.11
*CBS*	0.904	*FOLR3*	2.3

## Data Availability

Not applicable.
